# Genome-Wide Analysis of Soybean Lateral Organ Boundaries Domain Gene Family Reveals the Role in *Phytophthora* Root and Stem Rot

**DOI:** 10.3389/fpls.2022.865165

**Published:** 2022-05-04

**Authors:** Siqi Feng, Jinxia Shi, Yongkang Hu, Die Li, Liang Guo, Zhibo Zhao, Gang-Seob Lee, Yongli Qiao

**Affiliations:** ^1^Department of Plant Pathology, College of Agriculture, Guizhou University, Guiyang, China; ^2^Shanghai Key Laboratory of Plant Molecular Sciences, College of Life Sciences, Shanghai Normal University, Shanghai, China; ^3^National Institute of Agricultural Science, Jeonju, South Korea

**Keywords:** LBD gene family, phylogenetic analysis, *Phytophthora* root and stem rot, plant defense, soybean

## Abstract

The plant-specific lateral organ boundaries (LOB) domain (LBD) proteins, a family of transcription factors, play important roles in plant growth and development, as well as in responses to various stresses. However, little is known about the functions of *LBD* genes in soybean (*Glycine max*). In this study, we investigated the evolution and classification of the LBD family in soybean by a phylogenetic tree of the *LBD* gene family from 16 species. Phylogenetic analysis categorized these proteins into two classes (Class I and Class II) with seven subgroups. Moreover, we found that all the 18 *LBD* ancestors in angiosperm were kept in soybean, common bean genomes, and genome-wide duplication, suggesting the main force for the expansion of LBD from common bean to soybean. Analysis of gene expression profiling data indicated that 16 *GmLBD* genes were significantly induced at different time points after inoculation of soybean plants (cv. Huachun 6) with *Phytophthora sojae* (*P. sojae*). We further assessed the role of four highly upregulated genes, *GmLBD9*, *GmLBD16*, *GmLBD23*, and *GmLBD88*, in plant defense in soybean hairy roots using the transient overexpression and knockdown assays. The results showed that *GmLBD9* and *GmLBD23* negatively regulate plant immunity against *P. sojae*, whereas *GmLBD16* and *GmLBD88* positively manipulate plant immunity against *P. sojae*. Collectively, our findings expand our knowledge of the origin and evolution of the *GmLBD* gene family in soybean and promote the potential application of these genes in soybean genetic improvement.

## Introduction

The lateral organ boundaries (LOB) domain (LBD) proteins are a family of a plant-specific transcription factor with a characteristic N-terminal LBD (Iwakawa et al., 2002). So far, LBD has only been identified in the plant genome, indicating that this unique plant gene family only regulates the plant’s developmental process (Shuai et al., 2002). Following the identification of LBD in *Arabidopsis*, LBDs have also been found in many other plant species, such as *Oryza sativa*, *Malus domestica*, *Zea mays*, and *Vitis vinifera*. The number of LBD family members identified in different plant genomes greatly varied ranging from < 10 to > 100 (Yang et al., 2006, 2016, 2017; Wang et al., 2013; Zhang et al., 2014; Cao et al., 2016; Luo et al., 2016; Gombos et al., 2017; Grimplet et al., 2017; Lu et al., 2018).

According to the structural characteristics of the LOB domain, the LBD family can be divided into two subclasses, namely, Class I and Class II (Shuai et al., 2002; Matsumura et al., 2009). Class I family members encode proteins containing two conserved motifs in the LOB domain, namely, a CX_2_CX_6_CX_3_C zinc finger-like coiled-coil motif and an LX_6_LX_3_LX_6_L leucine zipper-like motif (Shuai et al., 2002; Lee et al., 2009), while family members in Class II contain only a zinc finger-like motif, lacking a leucine zipper-like motif. Due to the incomplete LBD sequence and unstable structure in Class II LBDs, the majority of LBDs belong to Class I (Majer and Hochholdinger, 2011). In model plant *Arabidopsis*, among 42 LBD family numbers, 36 genes are classified into Class I and 6 genes into Class II (Iwakawa et al., 2002). Similarly, among 90 LBDs from *Glycine max* (*G. max*, Soybean), 74 GmLBDs are classified into Class I and only 16 GmLBDs into Class II (Yang et al., 2017).

Many researches about the LBD family evolution have been performed to explore how this family was classified and originated. Chanderbali et al. (2015) found that LBD might be originated during the early evolution of charophyte algae when they constructed a comprehensive phylogenetic tree of LBD from 307 species, including angiosperms, gymnosperms, monilophytes, lycophytes, liverworts, hornworts, and charophyte algae. No LBDs were identified in *Chlamydomonas reinhardtii* and *Volvox carteri*, but several members can be found in Charales species, which suggested that the LBD family already existed before algae and evolved through extensive expansion during land plant diversification (Tang, 2013). Coudert et al. (2013) investigated the gene collinearity of 11 representative plant species and retraced *LBD* ancestor genes for early land plants, seed plants, and angiosperms, respectively, which lays the foundation for illustrating the diversification of the LBD family. For the study about the classification of the LBD family, Class I and Class II gene families can be clearly divided into many species due to the obvious sequencing difference in the LOB domain. Further subdivisions of Class I members revealed highly dynamic patterns in different species. In *Arabidopsis*, Class I LBDs were divided into four subgroups. Five subgroups were classified as rice Class I members. The inconsistent subgroup number might be due to the limited gene diversity in a single plant genome or massive gene duplications. Recently, extensive efforts have been exerted to analyze the phylogenetic distribution of Class I members from multiple species and concluded that the diversification in Class I established five branches, namely, Class IA, IB, IC1/ID, IC2, and IE (Coudert et al., 2013; Chanderbali et al., 2015). And this classification has successfully been proved in other studies and is regarded as the classification standard of Class I LBD members (Yu et al., 2020; Zhang et al., 2020).

Lateral organ boundaries domain proteins were initially believed to play roles in lateral organ development and then were demonstrated to play versatile functions in subsequent functional studies. LBD members in Class I are mostly involved in plant development, such as lateral organ development (Majer and Hochholdinger, 2011; Xu et al., 2016) and auxin signal transduction cascade (Liu et al., 2005; Lee et al., 2015). Members in Class II are involved in metabolisms, such as repressors of anthocyanin synthesis and N availability signals (Rubin et al., 2009). From expression profiles of *LBD* family genes in *Arabidopsis*, some *LBD* genes that belong to Class II were responsive to multiple pathogens, suggesting their functions in plant defense responses (Thatcher et al., 2012a). Further functional analysis showed that AtLBD20 showed resistance suppression against *Fusarium oxysporum* (*F. oxysporum*) infection since overexpression of AtLBD20 in roots promoted the colonization of *F. oxysporum* (Thatcher et al., 2012b). Expression pattern of *GmLBD* genes after pathogens infection indicated that several *GmLBDs* were induced in the root and hypocotyl after *Bradyrhizobium japonicum* and *P. sojae* mycelia infection (Yang et al., 2017). However, the detailed characterization of GmLBD functions in plant immunity remains unexplored.

In this study, we reconstructed the phylogenetic tree of the LBD gene family from 16 representative genome-available plant species and then compared the evolutionary patterns between soybean and common bean. In addition, based on the expression patterns of *P. sojae* infection, four GmLBDs were selected for further functional analysis to examine their roles in plant immunity.

## Results

### Identification and Phylogenetic Analysis of LBD Genes in 16 Plant Species

Soybean *LBD* genes (*GmLBDs*) have been previously identified (Yang et al., 2017). To further understand the functions of *GmLBDs* in their origination, classification, and even the evolutionary relationship with other species in *Leguminosae*, we first identified LBD family members in the *Phaseolus vulgaris* genome (*P. vulgaris*, common bean), a species with a relatively close evolutionary relationship with soybean in *Leguminosae*, and in the *Cucumis sativus* genome (*C. sativus*, cucumber), an eudicot species. A local BLASTP search was carried out using 42 known *Arabidopsis* LBD proteins as the query in common bean and cucumber genomes in the NCBI database. Subsequently, all potential LBD protein sequences were further verified by domain analysis using Pfam (LOB domain, DUF260, Pfam number: Pfam03195). As a result, a total of 42 CsLBDs in cucumber and 50 PvLBDs in common bean were finally identified. CsLBDs and PvLBDs were named according to the order of locations on the chromosomes (Supplementary Tables 1, 2).

To further improve the understanding of the phylogenetic classification and evolution of the LBD family in the soybean genome, a comprehensive phylogenetic tree was constructed using 788 amino acid sequences of LBD protein from 16 plant species, including one species each of green alga, moss, fern, and basal angiosperm; eight species in eudicots; and four species in monocots (Supplementary Table 3). The 788 amino acid sequences of LBD protein contained 696 known LBD proteins from 14 species and 92 LBD proteins that were newly identified in this study (Yang et al., 2017; Zhang et al., 2020).

**FIGURE 1:**
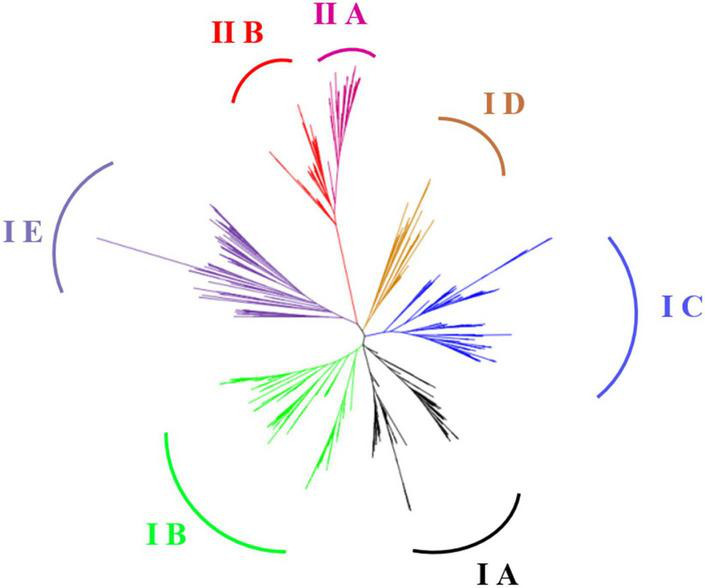
Phylogenetic analysis of LBD proteins in 16 plant species. In total, 788 full-length amino acid sequences in LBD proteins were aligned with Clustal X. Phylogenetic tree was constructed with ML (maximum-likelihood) method in MEGA X and 100 times of bootstrap replicates. Different subgroups in the phylogenetic tree are labeled with different colors.

Phylogenetic analysis showed that all LBD proteins were classified into two classes (Class I and Class II); Class I is further divided into five subgroups, namely, Class IA, Class IB, Class IC, Class ID, and Class IE, whereas Class II is divided into Class IIA and Class IIB (Figure 1), which is consistent with the previous results (Tang, 2013; Chanderbali et al., 2015; Zhang et al., 2020). Among the 90 GmLBDs, 74 were clustered into Class I, with 19 in Class IA, 25 in Class IB, 19 in Class IC, 4 in Class ID, and 7 in Class IE, while 16 GmLBDs were clustered into Class II.

### Evolutionary Relationship of *LBD* Genes Between Soybean and Common Bean

**FIGURE 2:**
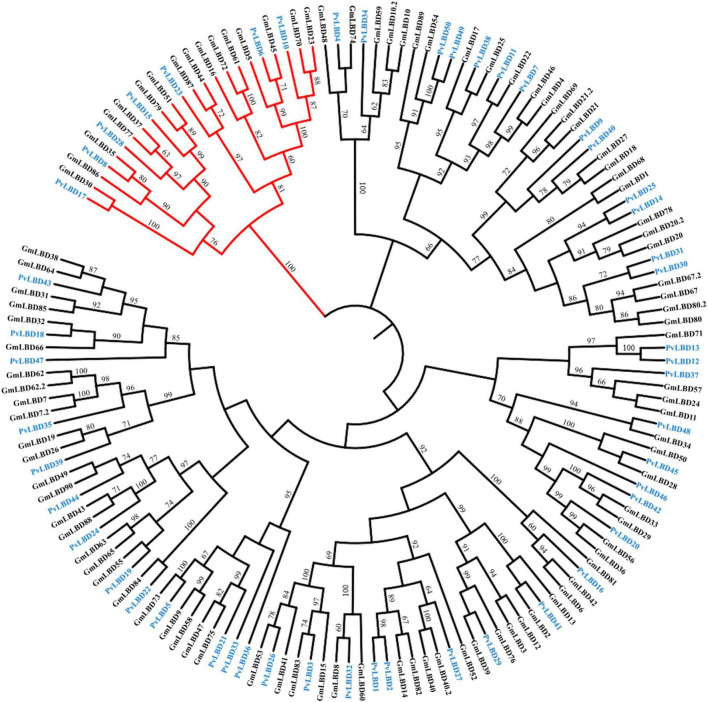
Phylogenetic analysis of LBD proteins in soybean and common bean. The phylogenetic tree was constructed according to the same method. Class II LBD family members were shown in red branches. GmLBDs and PvLBDs were marked with green and blue protein names, respectively. Bootstrap values of more than 60 are represented on each node.

Given that soybean and common bean have been demonstrated with a close genetic relationship (Vlasova et al., 2016) and both of them are important cash crops. We, therefore, constructed a phylogenetic tree between soybean and common bean to explore the evolutionary relationship of *LBD* genes in these two genomes using full-length protein sequence. Phylogenetic analysis showed that the homologous relationships between *GmLBDs* and *PvLBDs* were obviously observed since almost all clades were included by one *PvLBDs* and one or two *GmLBDs* (Figure 2). The homologous relationships were inspected by checking *GmLBDs* and *PvLBDs* in the same clades and summarized (refer to Table 1). Intriguingly, we found that a total of 38 homologous gene groups were detected, including all *PvLBDs* and 91% of *GmLBDs* (82/90), suggesting that the gene duplication in soybean *LBDs* is another character. In around 70% of clades (25 in 42 clades), one *PvLBD* and two *GmLBDs* were closely clustered into one new clade. In the homologous gene group summary, 25 (65%) groups showed the gene ratio of *PvLBDs*:*GmLBDs* as 1:2, i.e., one *PvLBD* has two *GmLBDs* orthologs.

**TABLE 1:** Summary of *GmLBD* and *PvLBD* synteny gene pairs derived from phylogenetic analysis.

*PvLBD* genes	*GmLBD* genes	Ratio (Pv vs Gm)
*PvLBD17*	*GmLBD30*	1:1
*PvLBD8*	*GmLBD86, GmLBD35*	1:2
*PvLBD28*	*GmLBD77, GmLBD37*	1:2
*PvLBD15*	*GmLBD79, GmLBD51*	1:2
*PvLBD23*	*GmLBD87, GmLBD44*	1:2
*PvLBD6*	*GmLBD5, GmLBD45*	1:2
*PvLBD10*	*GmLBD70, GmLBD23*	1:2
*PvLBD4*	*GmLBD48, GmLBD74*	1:2
*PvLBD34*	*GmLBD59, GmLBD10*	1:2
*PvLBD50, PvLBD49*	*GmLBD65, GmLBD54, GmLBD89*	2:3
*PvLBD38*	*GmLBD17, GmLBD25*	1:2
*PvLBD11*	*GmLBD22*	1:1
*PvLBD7*	*GmLBD4, GmLBD46*	1:2
*PvLBD9, PvLBD40*	*GmLBD27, GmLBD18, GmLBD69, GmLBD21*	2:4
*PvLBD25, PvLBD14*	*GmLBD78, GmLBD20*	2:2
*PvLBD31, PvLBD30*	*GmLBD67, GmLBD80*	2:2
*PvLBD12, PvLBD13*	*GmLBD71*	2:1
*PvLBD37*	*GmLBD57, GmLBD24*	1:2
*PvLBD48*	*GmLBD34*	1:1
*PvLBD45*	*GmLBD50, GmLBD28*	1:2
*PvLBD46, PvLBD42*	*GmLBD33, GmLBD29*	2:2
*PvLBD20*	*GmLBD56, GmLBD36*	1:2
*PvLBD16*	*GmLBD81, GmLBD6, GmLBD42*	1:3
*PvLBD41*	*GmLBD13, GmLBD2*	1:2
*PvLBD29*	*GmLBD76, GmLBD39*	1:2
*PvLBD27*	*GmLBD52, GmLBD40*	1:2
*PvLBD1, PvLBD2*	*GmLBD14, GmLBD82*	2:2
*PvLBD32*	*GmLBD8, GmLBD60*	1:2
*PvLBD3*	*GmLBD15, GmLBD83*	1:2
*PvLBD26*	*GmLBD41, GmLBD53*	1:2
*PvLBD33, PvLBD36, PvLBD21, PvLBD5*	*GmLBD75, GmLBD47, GmLBD58, GmLBD9, GmLBD73*	4:5
*PvLBD22*	*GmLBD84*	1:1
*PvLBD19*	*GmLBD55*	1:1
*PvLBD24*	*GmLBD88, GmLBD43*	1:2
*PvLBD44*	*GmLBD90, GmLBD49*	1:2
*PvLBD39*	*GmLBD26, GmLBD19*	1:2
*PvLBD35*	*GmLBD7, GmLBD62*	1:2
*PvLBD47, PvLBD18, PvLBD43*	*GmLBD66, GmLBD32, GmLBD85, GmLBD31, GmLBD64, GmLBD38*	3:6
	GmLBD16*, GmLBD72*, GmLBD61*	–
	GmLBD68*, GmLBD1*	–
	GmLBD3*, GmLBD12*	–
	*GmLBD63* *	–

*indicated GmLBDs failing to find paralogs in common bean.

**FIGURE 3:**
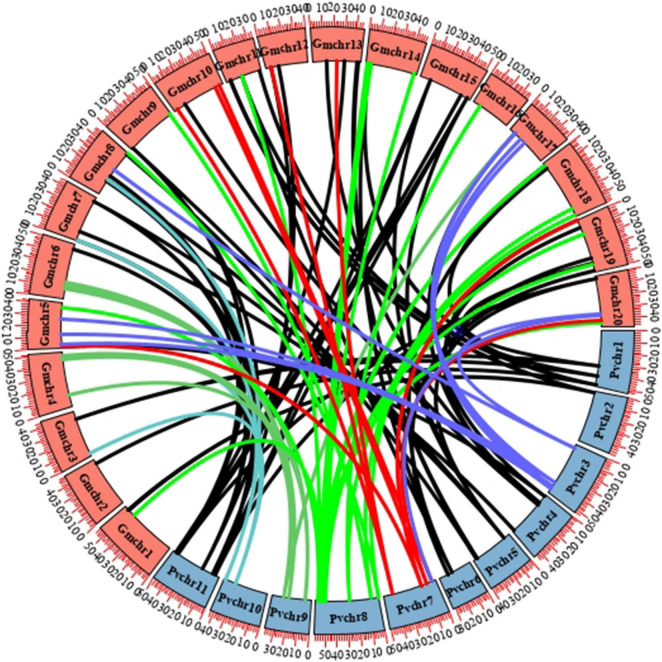
Synteny analysis of *LBD* genes in soybean and common bean. Circular collinearity analysis of *LBD* genes in soybean and common bean genomes. *GmLBDs* and *PvLBDs* were mapped to their corresponding chromosomal locations and represented in a circular diagram using Circos. Colored lines connect the pairs of orthologous *LBD* genes in the syntenic blocks of these two genomes. Soybean and common bean chromosomes are denoted as red and blue boxes, respectively.

Soybean experienced one-time independent whole-genome duplication (WGD) compared with common bean, and they diverged only 19.2 million years ago, a relatively short time compared with other legume sister species. To verify the mechanism of gene duplication between soybean and common bean *LBD* genes, syntenic maps of *LBD* homologs in these two genomes were built; *GmLBD89* and *GmLBD90* were excluded in syntenic analysis because of the unassembled genome locations (Figure 3). As a result of 90 *GmLBDs* and 50 *PvLBDs*, 112 collinear gene pairs were detected and merged into 38 collinear groups. The collinear groups contain 93% (82/88) of *GmLBDs*, and 100% (50/50) of *PvLBDs* genes, which is perfectly consistent with the homologous gene groups summary (Table 1). Interestingly, all these collinear gene pairs contained about 1.62 pairs of conserved genes on average, which is also consistent with the summary of paralog ratios between these two species (Table 1). The syntenic analysis results again proved the close evolutionary relationship of these two species and suggested that WGD might be the main force for LBDs expansion in the soybean genome.

A total of 18 *AtLBD* ancestries in angiosperms have been previously retraced based on the gene collinearity investigation and phylogenetic relationships (Kong et al., 2017). To further characterize the evolutionary patterns, we also tried to trace the ancestries in *GmLBDs* and *PvLBDs*. Interestingly, our data exhibited that *GmLBDs* and *PvLBDs* can be detected in all 18 *AtLBDs* ancient lineages (Table 2), suggesting that no ancestor genes were lost in soybean and common bean genomes. In each *AtLBD* ancient lineage, 2–10 *GmLBD* and 1–6 *PvLBD* paralogs were presented. In most ancient lineage, the number of *GmLBDs* was much more than that of *AtLBDs*, such as in lineage 2, *AtLOB* and *AtLBD25* vs 10 *GmLBDs* and in lineage 11, *AtLBD3* vs 6 *GmLBDs*, which indicated the extensive expansion of *GmLBDs* in these ancient lineages. However, in the common bean genome, no obvious gene expansion was found except in lineages 2, 9, and 11. The decrease of gene number was also found in some ancient lineages for both *GmLBDs* and *PvLBDs*, such as in ancient lineage 8, 4 *AtLBDs* vs 2 *GmLBDs* vs 1 *PvLBD* and in ancient lineage 15, 6 *AtLBDs* vs 3 *GmLBDs* vs 2 *PvLBD*. The ancestry retracement in soybean and common bean demonstrated that LBD is reluctant to be lost and the similar expansion and decrease patterns in some ancient lineages between soybean and common bean mean that they might suffer from parallel evolution.

**TABLE 2:** Summary of *GmLBDs* and *PvLBDs* presented in 18 *AtLBD* ancestral lineages.

No.	*AtLBD* ancestral lineage	*GmLBDs*	Numbers	*PvLBDs*	Numbers
1	*AtLBD21*	*GmLBD48, GmLBD74, GmLBD59, GmLBD10*	4	*PvLBD4, PvLBD34*	2
2	*AtLOB, AtLBD25*	*GmLBD27, GmLBD18, GmLBD69, GmLBD21, GmLBD68, GmLBD1, GmLBD78, GmLBD20, GmLBD67, GmLBD80*	10	*PvLBD40, PvLBD9, PvLBD25, PvLBD14, PvLBD31, PvLBD30*	6
3	*AtLBD6*	*GmLBD54, GmLBD89*,	2	*PvLBD50, PvLBD49*	2
4	*AtLBD10, AtLBD32, AtLBD35, AtLBD36*	*GmLBD22, GmLBD25, GmLBD17, GmLBD46, GmLBD4, GmLBD71, GmLBD11, GmLBD57, GmLBD24*	9	*PvLBD11, PvLBD38, PvLBD7, PvLBD13, PvLBD12, PvLBD37*	6
5	*AtLBD20*	*GmLBD6, GmLBD42, GmLBD81*	3	*PvLBD16*	1
6	*AtLBD18, AtLBD19, AtLBD30, AtLBD31*	*GmLBD13, GmLBD2, GmLBD12, GmLBD3, GmLBD76, GmLBD39*	6	*PvLBD41, PvLBD29*	2
7	*AtLBD16*	*GmLBD15, GmLBD83, GmLBD53, GmLBD41*	4	*PvLBD3, PvLBD26*	2
8	*AtLBD33, AtLBD14, AtLBD17, AtLBD29*	*GmLBD60, GmLBD8*	2	*PvLBD32*	1
9	*AtLBD12*	*GmLBD58, GmLBD9, GmLBD73, GmLBD75, GmLBD47*	5	*PvLBD36, PvLBD33, PvLBD5, PvLBD21*	4
10	*AtLBD23, AtLBD24*	*GmLBD11, GmLBD57, GmLBD24*	3	*PvLBD37*	1
11	*AtLBD3*	*GmLBD64, GmLBD38, GmLBD85, GmLBD31, GmLBD66, GmLBD32*	6	*PvLBD43, PvLBD47, PvLBD18*	3
12	*AtLBD4, AtLBD1, AtLBD11*	*GmLBD63, GmLBD65, GmLBD55, GmLBD49, GmLBD90, GmLBD84*	6	*PvLBD24, PvLBD19, PvLBD44, PvLBD22*	4
13	*AtLBD13, AtLBD15*	*GmLBD26, GmLBD19, GmLBD7, GmLBD62*	4	*PvLBD39, PvLBD35*	2
14	*AtLBD27, AtLBD34*	*GmLBD33, GmLBD66, GmLBD29*	3	*PvLBD46*	1
15	*AtLBD2, AtLBD5, AtLBD8, AtLBD9, AtLBD26, AtLBD28*	*GmLBD50, GmLBD28, GmLBD34*	3	*PvLBD45, PvLBD48*	2
16	*AtLBD7, AtLBD22*	*GmLBD33, GmLBD29, GmLBD5, GmLBD6, GmLBD36*	5	*PvLBD20, PvLBD42*	2
17	*AtLBD37, AtLBD38, AtLBD39*	*GmLBD44, GmLBD87, GmLBD16, GmLBD72, GmLBD61, GmLBD23, GmLBD70, GmLBD45, GmLBD5*	9	*PvLBD23, PvLBD10, PvLBD6*	3
18	*AtLBD40, AtLBD41, AtLBD42*	*GmLBD79, GmLBD51, GmLBD77, GmLBD37, GmLBD35, GmLBD86, GmLBD30*	7	*PvLBD15, PvLBD28, PvLBD8, PvLBD17*	4

### Expression Profiles of *GmLBD* Genes During *P. sojae* Infection

LBD proteins have been reported to play important roles in controlling plant growth and development and also in responding to stress, such as pathogen infection (Iwakawa et al., 2002; Shuai et al., 2002; Feng et al., 2012). To further examine the potential roles of GmLBD proteins in plant immunity, especially in response to *P. sojae* infection, some *GmLBD* candidate genes were first identified. Based on previous studies, there are some LBD proteins, which have previously been characterized to involve in plant immune response or upregulated by pathogen infection. *AtLBD20* in *Arabidopsis* and *CsLOB1* in *Citrus sinensis* were found to be involved in plant immunity response to the pathogen (Thatcher et al., 2012b; Hu et al., 2014). So a BLASTP search was performed against the soybean genome database^1^ using AtLBD20 and CsLOB1 as queries and identified 6 GmLBD homologs. In total, 13 *GmLBDs* were reported to be highly induced in responses to biotic stresses (Yang et al., 2017). Accordingly, a total of 19 *GmLBDs* genes were selected as candidates for further functional characterization (Supplementary Table 4).

**FIGURE 4:**
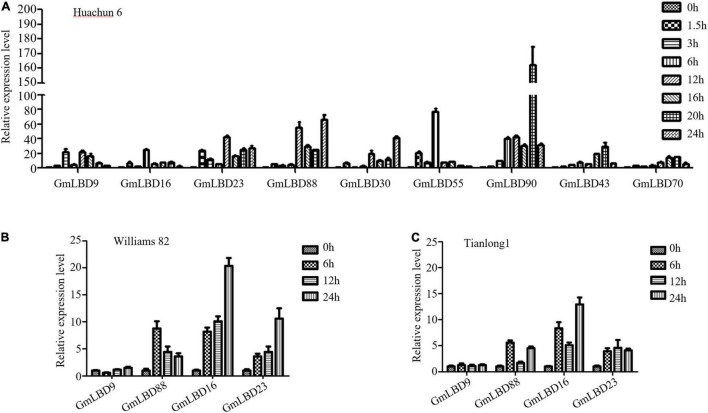
Expression profiles of *GmLBD* genes in Huanchun 6, Williams 82, and Tianlong 1 cultivars upon *P. sojae* infection.**(A)** Soybean hairy roots were collected at 0, 1.5, 3, 6, 12, 16, 20, and 24 h after *P. sojae* strain P6497 infection. Total RNA was extracted and expression profiles of 9 *GmLBD* genes at various time points during infection were determined by qRT-PCR. The Soybean *GmCYP2* gene was used as an internal control. Error bars indicate three biological replicates. Soybean hairy roots of Williams 82 **(B)** and Tianlong 1 **(C)** were collected at 0, 6, 12, and 24 h after *P. sojae* strain P6497 infection. Total RNA was extracted and expression profiles of 4 *GmLBD* genes at various time points during infection were determined by qRT-PCR. The Soybean *GmCYP2* gene was used as an internal control. Error bars indicate three biological replicates.

To determine whether these candidate genes play roles in plant defense response against *P. sojae* attack, the expression patterns of 19 *GmLBDs* genes upon *P. sojae* infection were examined. The quantitative reverse-transcription PCR (qRT-PCR) was performed using RNA that was extracted from hairy roots of soybean susceptible species Huachun 6 and collected at different time points [0, 1.5, 3, 6, 12, 20, and 24 h after infection (hpi)] after *P. sojae* infection. The results showed that 16 out of 19 genes were successfully amplified by qRT-PCR, and 15 *GmLBD*s were found to be induced in the early infection period except for *GmLBD37* when compared with the uninfected samples (Figure 4). Among them, 9 *GmLBD* genes (*GmLBD9*, *GmLBD16*, *GmLBD23*, *GmLBD88*, *GmLBD30*, *GmLBD55*, *GmLBD90*, *GmLBD43*, and *GmLBD70*) were considered as highly upregulated genes, since the highest expression levels were increased at least 20-fold, especially for *GmLBD90*, which was induced more than 50-fold from 6 to 24 hpi and reached the highest expression level at 20 hpi for up to 150-fold (Figure 4A). The remaining six *GmLBD* genes (*GmLBD31*, *GmLBD45*, *GmLBD51*, *GmLBD49*, *GmLBD59*, and *GmLBD63*) were upregulated approximately 2–6 times (Supplementary Figure 1).

To further confirm the expression profiles of *GmLBDs* upon *P. sojae* infection, four *GmLBD* genes (*GmLBD9*, *GmLBD16*, GmLBD23, and GmLBD88), which showed highly induced expression patterns in Huachun 6, were selected to investigate their expressions in other soybean cultivars. We chose two soybean cultivars (Williams 82 and Tianlong 1) for further determining the expression pattern of these four genes. Williams 82, with a resistance gene (*Rps1k*) (Mideros et al., 2007), is known to be resistant to *P. sojae* strain P6497, and Tianlong 1 showed moderate resistance against P6497 than susceptible species Huachun 6 (personal communication). Compared to that in Huachun 6, *GmLBD88*, *GmLBD16*, and *GmLBD23* genes also showed the upregulated expression patterns in Williams 82 and Tianlong 1 upon P6497 inoculation, while no clear expression change was detected in *GmLBD9* (Figures 4B,C).

To further elucidate the function of these genes in soybean, their tissue-specific expression patterns were also examined in soybean roots, stems, and leaves by qRT-PCR. The results showed that these four genes were ubiquitously expressed in all plant organs tested, with the highest expression level in roots for *GmLBD9*, *GmLBD16*, and *GmLBD23* genes and the highest expression level in stems for the *GmLBD88* gene (Supplementary Figure 2). Given that most of the candidate *GmLBD* genes showed induced expression upon *P. sojae* infection, we inferred that they might play important roles in early soybean defense response.

### *GmLBD* Genes Closely Associated With Soybean Immunity Against *P. sojae* Infection

**FIGURE 5:**
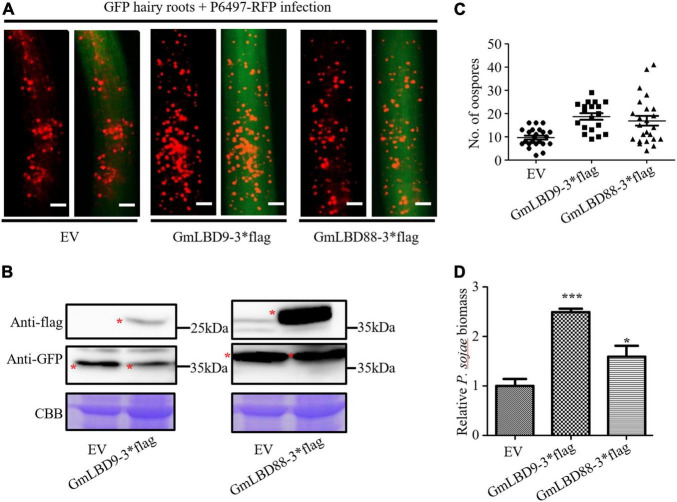
Overexpression of *GmLBD9* and *GmLBD88* enhanced *P. sojae* infection. **(A)** Soybean hairy roots overexpressing EV, GmLBD9-3*flag, and GmLBD88-3*flag were selected based on the green fluorescence and then inoculated with *P. sojae* strain P6497-RFP. *P. sojae* oospores were observed at 48 hpi under a fluorescence microscope. Scale bars represent 0.28 mm. **(B)** Expression of recombinant proteins EV, GmLBD9-3*flag, and GmLBD88-3*flag was detected in western blot. Protein gel was stained with Coomassie blue as the loading control. **(C)** The number of oospores was counted. **(D)** Relative biomass of *P. sojae* was determined by qPCR at 48 hpi, and standard errors from three replicates are shown (*P < 0.05; ^***^P < 0.001; one-way ANOVA).

**FIGURE 6:**
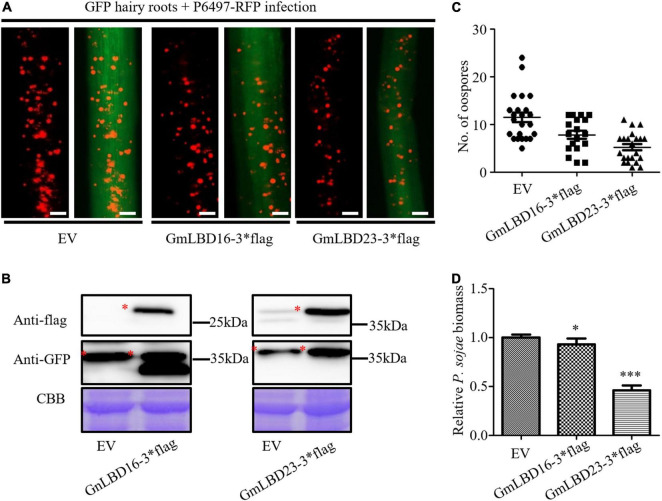
Overexpression of *GmLBD16* and *GmLBD23* suppressed *P. sojae* infection.**(A)** Soybean hairy roots overexpressing EV or GmLBD16-3*flag and GmLBD23-3*flag were selected based on the green fluorescence and then inoculated with *P. sojae* strain P6497-RFP. *P. sojae* oospores were observed at 48 hpi under a fluorescence microscope. Scale bars represent 0.28 mm. **(B)** Expression of recombinant proteins EV, GmLBD16-3*flag, and GmLBD23-3*flag was detected in western blot. Protein gel was stained with Coomassie blue as the loading control. **(C)** The number of oospores was counted. **(D)** Relative biomass of *P. sojae* was determined by qPCR at 48 hpi, and standard errors from three replicates are shown (*P < 0.05; ^***^P < 0.001; one-way ANOVA).

To investigate the functions of *GmLBD* genes in soybean immunity, four *GmLBD* genes (*GmLBD9*, *GmLBD16*, *GmLBD23*, and *GmLBD88*) showed remarkably upregulated expression in Huachun 6 were chosen to reveal how they regulate soybean immunity through the transient overexpression and knockdown assays. *GmLBD9*, *GmLBD16*, *GmLBD23*, and *GmLBD88* were first transiently overexpressed in soybean hairy roots by *Agrobacterium*-mediated transformation, and then those transformed hairy roots were inoculated with *P. sojae* strain P6497-RFP. We discovered that more *P. sojae* oospores can be observed in hairy roots expressing *GmLBD9* and *GmLBD88* at 48 hpi in relative to those hairy roots expressing empty vector (EV) by microscope observation (Figure 5A). Consistently, oospores and biomass of *P. sojae* were much higher in soybean hairy roots inoculated with *GmLBD9* and *GmLBD88* than in roots inoculated with the EV control (Figures 5C,D); these data indicate that the expression of *GmLBD9* and *GmLBD88* could promote the colonization of *P. sojae* in soybean hairy roots. Interestingly, we found that the individual expression of *GmLBD16* and *GmLBD23* inhibited *P. sojae* infection in soybean hairy roots, showing fewer oospores and lower relative biomass of *P. sojae* in transiently expressing hairy roots (Figures 5A, 6C,D). Moreover, the expression of those four recombinant proteins was confirmed by western blot, respectively (Figures 5B, 6B). Overall, these results suggest that *GmLBD9* and *GmLBD88* may be the negative immune regulators for soybean resistance against *P. sojae*, while *GmLBD16* and *GmLBD23* may be the positive immune regulators.

**FIGURE 7:**
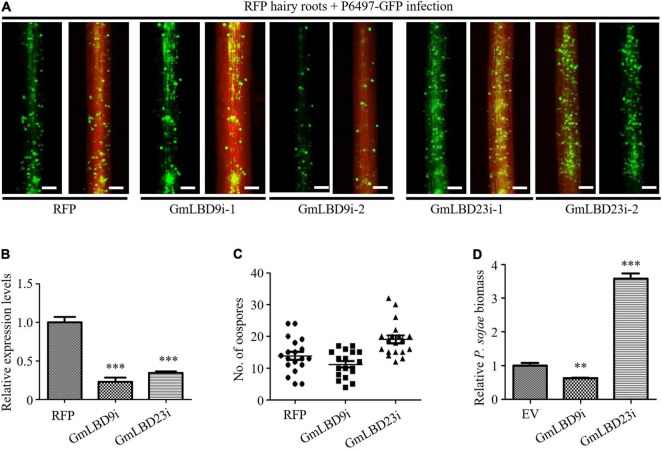
Silencing of *GmLBD9* and *GmLBD23* showed opposite roles in soybean immunity to *P. sojae* infection.**(A)** Hairy roots expressing GmLBD9- and GmLBD23-silenced constructs were selected based on the red fluorescence and then inoculated with P6497-GFP. *P. sojae* oospores were observed at 48 hpi under a fluorescence microscope. Scale bars represent 0.28 mm. **(B)** Relative expression of *GmLB9* and *GmLBD23* in soybean hairy roots was confirmed by qRT-PCR. *GmCYP2* gene was used as an internal control. **(C)** The number of oospores was observed under fluorescence microscopy and counted. **(D)** Relative biomass of *P. sojae* was determined by qPCR at 48 hpi. Error bars indicate three biological replicates (^**^P < 0.01; ^***^P < 0.001; one-way ANOVA).

These data prompted us to further verify their roles in soybean *Phytophthora* root and rot. Two *GmLBDs* (*GmLBD9* and *GmLBD23*) were selected to perform transient silencing in soybean hairy roots by RNA interference (RNAi) technique. *GmLBD9* and *GmLBD23* have been proved to be negative and positive immune regulators in soybean immunity, respectively. To specifically silence *GmLBD9* or *GmLBD23*, approximately 200–300 bp of 5′- or 3′-end UTR fragments were cloned into pK7GWIWG2D vector to generate an RNAi recombinant construct. And then, these constructs were introduced into soybean hairy roots by *Agrobacterium*-mediated transformation. Each RNAi construct successfully silenced these targets as shown by qRT-PCR analysis and revealed that the expression of *GmLBD9* and *GmLBD23* were obviously reduced by 70–80% in silencing hairy roots (Figure 7B). Subsequently, these silenced hairy roots were challenged with *P. sojae* strain P6497-GFP. The results displayed that *GmLBD9*-silenced hairy roots showed less oospores (Figures 7A,C) and lower relative biomass of *P. sojae* than those hairy roots induced by EV (Figure 7D). Together with the above data, our results further indicated that the *GmLBD9* gene negatively regulates soybean immunity against *P. sojae* infection. As such, the infection on *GmLBD23*-silenced roots exhibited that much more oospores were observed through fluorescence microscope observation (Figure 7A). Quantification of oospore number and biomass also proved that silencing of *GmLBD9* promoted colonization of *P. sojae* in soybean hairy roots (Figures 7C,D), indicating that the *GmLBD9* gene positively manipulates soybean immunity against *P. sojae* infection. Taken together, our data suggest that *GmLBD9* and *GmLBD88* are two negative immune regulators and *GmLBD16* and *GmLBD23* are two positive regulators of plant immunity against *P. sojae* infection.

## Discussion

As a plant-specific gene family, LBD family proteins have drawn many researchers’ attention to explore their phylogenetic diversification, origination, and even functional characteristics by genome-wide analysis (Wang et al., 2013; Zhang et al., 2014; Yang et al., 2016). In this study, amino acid sequences of LBD members from 16 representative species, including green alga, basal angiosperm, monocots, and dicots, were collected, and a comprehensive phylogenetic tree was constructed (Figure 1). We mainly focused on the evaluation of LBD family members in soybean and common bean. Our data have shown that all 18 ancient gene lineages for angiosperms were preserved in soybean and common bean LBD family members. This is consistent with the divergence of the LBD family in early land plants, seed plants, and angiosperm. In early land plants, 7 ancient genes are deduced and kept in a stable amount. Also in angiosperm genomes, 18 major lineages can be detected in rice and *Arabidopsis* genomes (Kong et al., 2017). Above all the results suggested that LBDs were reluctant to be lost during evolution. Moreover, it was supposed that the additional WGD events that happened in the soybean genome were probably the major driving force behind the substantial gene content increase due to the ratio of *LBD* ortholog numbers and synteny analysis between soybean and common bean. Similar results were also obtained by another gene family evolutionary analysis. Wu et al. investigated WRKY transcription factors in common beans and they deduced that it was the result of genome duplication of the two WRKY transcription factors in the soybean genome rather than in the common bean genome (Wu et al., 2017).

Although the expression dataset of *GmLBDs* in various tissues and under biotic and abiotic stresses, including pathogen infection, is available online (Yang et al., 2017), functional characterization of *GmLBDs* in plant immunity has not been previously documented. In this study, four selected *GmLBDs* were significantly induced on *P. sojae* infection; however, *GmLBD9* and *GmLBD88* showed opposite roles with *GmLBD16* and *GmLBD23* in the subsequent functional analysis (Figures 5–7). It might be caused by two possible reasons: one is that the gene responses might be tissue, growth stage, or genotype-specific. In this study, three species showing different resistance to *P. sojae* strain P6497 were included in examining gene expression patterns. *GmLBD9* was highly induced in Huachun 6 but showed no change in Williams 82 and Tianlong 1. Gene expression between four chickpea genotypes, including resistant, moderately resistant, susceptible, and wild relative genotype, was different when these four genotypes were challenged by ascochyta blight (Coram and Pang, 2006). Expression comparison of responses to volatiles in *Arabidopsis* revealed that genes involved in flavonoid biosynthesis were downregulated in leaves and upregulated in roots, photosynthesis genes were impressed in the seeding stage and induced at the mature stage (Hao et al., 2016). Therefore, the gene expression profile is just a useful tool providing us with the potential candidates for further functional validation since it has fast, convenient, and high throughput. The other reason is that it is common that these genes were regulated by diverse pathways. *ShARPC3* can be highly induced during an incompatible and compatible interaction against On-Lz infection and finally turned out to be a positive regulator of plant immunity due to its overexpression inducing rapid hypersensitive cell death and reactive oxygen generation (Sun et al., 2019). EDS1-interacting J protein 1 (EIJ1) is proved as an EDS1-dependent negative regulator of innate plant immunity with significant induction by the treatment with *Pst DC3000* or SA (Liu et al., 2021).

In summary, 788 LBDs from 16 species, including 90 from soybean and 50 from common bean, were used to perform an extensive phylogenetic analysis of LBD proteins. Phylogenetic analysis categorized these proteins into two groups, namely, Class I and Class II, and Class I was further classified into five subgroups. None of the ancestor genes were lost in the soybean and common bean genomes in ancestor gene retracement. The evolutionary analysis indicated that the expansion of LBD numbers in the soybean genome was primarily driven by WGD. Based on the gene expression profiles on *P. sojae* infection, four *GmLBDs* were chosen for further functional characterization and discovered *GmLBD9* and *GmLBD88* function as negative immune regulators and *GmLBD16* and *GmLBD23* as positive immune regulators in plant immunity. So this study expands our knowledge of the origin and evolution of the GmLBD gene family in soybean and promotes the potential application of these genes in soybean genetic improvement.

## Materials and Methods

### Plant and Microbe Cultivation

Soybean cotyledons (Huachun 6, Williams 82 and Tianlong 1) were grown in a greenhouse at 25°C with a 16/8 h (light/dark) photoperiod. *P. sojae* strains, namely, P6497, P6497-RFP, and P6497-GFP, were routinely maintained on a 10% vegetable (V8) juice medium at 25°C in darkness.

### Identification of *LBD*s in Cucumber and Common Bean

To obtain cucumber and common bean LBD protein sequences, all known 43 *Arabidopsis* LBD protein sequences were used as a query to perform BLASTP with an *e*-value of 1 × e^––10^ against the protein sequences database of *C. sativus* and *P. vulgaris* (NCBI^2^). Redundant sequences that are partial or alternatively spliced sequences from the same locus were removed. Then conserved domain of LBDs (LOB domain, DUF260, Pfam number: Pfam03195) acquired from Pfam^3^ was used for a blast to identify CsLBDs and PvLBDs with DUF260 as a query (El-Gebali et al., 2019). Finally, each gene was named based on its location on the chromosome.

### Phylogenetic Analyses

To construct a phylogenetic tree of LBDs in 16 species, 788 full-length LBD protein sequences were aligned using the multi-sequence alignment program ClustalW. A phylogenetic tree was constructed with ML (maximum-likelihood) method in MEGA X and 100 times of bootstrap replicates. The phylogenetic tree was further manipulated by the program Interactive Tree of Life (iTOL^4^) (Zhu et al., 2019).

### Synteny Analyses

The chromosomal length and locations of each GmLBD and PvLBD were retrieved from the soybean genome database in SoyBase^5^ and common bean genome database in NCBI. Advanced Circos program in TBtools (Chen et al., 2020a) was used for collinearity analyses.

### Plasmid Construction

For overexpression assay in soybean hairy roots, fragments containing full-length CDS sequences were amplified with gene-specific primers (Supplementary Table 5) and then ligated into vector PFGC5941 by homologous recombination (Vazyme, C112-02-AB), which adds a C terminal FLAG tag. For gene silencing assay, fragments derived from the 5′ or 3′ UTR regions with 200–300 bp in length were amplified and then cloned into pK7GWIWG2D vectors with Gateway technology (Thermo Fisher Scientific, 12538120).

### Transformation of Soybean Cotyledons and *P. sojae* Infection Assays

Soybean seeds (Huachun 6) were sanitized with a mixture of 84 disinfectants and concentrated hydrochloric acid (96:4) and then grown on germination medium (Gm medium). After around 6 days of growth, cotyledons were removed from soybean seedings and cut a wound (around 0.3 cm in diameter and 0.2–0.3 cm in depth) close to the petiole with a sterile knife. *Agrobacterium rhizogenes* (*A. rhizogenes*) K599 cell suspensions were inoculated on the wound, and then cotyledons were continued growing on Murashige and Skoog medium (MS medium). Around 3–4 weeks later, soybean hairy roots overexpressing or silencing GmLBDs were observed by fluorescence microscopy and further confirmed by western blot or qRT-PCR. Selected overexpressing hairy roots and silencing hairy roots were infected with P6497-RFP and P6497-GFP in wet and dark conditions at 25°C for around 48 h, respectively. Around 3- to 5-day-old *P. sojae* hyphae grown in 10% of V8 medium were used for soybean hairy roots infection.

### RNA Extraction and Quantitative Reverse-Transcription PCR Analysis

Total RNA was isolated from hairy roots using TRIzol Reagent (TaKaRa, 9109). Isolated RNA samples were quantified using a NanoDrop spectrophotometer (Thermo Fisher Scientific, NanoDrop One) and then treated with DNase I (Thermo Fisher Scientific, AM2222) to remove any residual DNA contamination. In total, 1 μg of DNA-free RNA samples were converted to cDNA using a cDNA synthesis kit (Vazyme Biotech, R212-02-AF). First-strand cDNA was synthesized using HiScript II 1st Strand cDNA Synthesis Kit (+gDNA wiper) with Oligo-(dT) 23VN (Vazyme Biotech, R212-02-AF).

For measuring the transcript level of *GmLBD* genes from soybean during *P. sojae* infection, 1- or 2-week-old soybean (Huachun 6, Williams 82 and Tianlong 1) secondary roots was infected with R6497 and collected at different time points after infection. For measuring the transcript level of *GmLBDs* in different tissues, leaves, roots, and stems were sampled from 3- to 4-week-old soybean plants. Total RNA was extracted and used as a template for reverse transcription. To determine gene silencing efficiency, RNA was extracted from 3- to 4-week-old hairy roots induced by K599 containing PK7GWIWG2D (II) and PK7GWIWG2D (II)-*GmLBDs*. In all the quantitative PCR (qPCR) reactions, gene-specific primers (Supplementary Table 5) were designed specifically in 5′- or 3′-end UTR of genes avoiding the fragments for gene silencing. Then, qPCR was carried out using Maxima SYBR Green/ROX qPCR Master Mix (Vazyme Biotech, Q711-02-AA) (Zhang et al., 2019; Zhu et al., 2020). *GmCYP2* gene (Hu et al., 2009) was used as an internal reference. Three independent biological replicates were conducted for each treatment with similar results.

### DNA Extraction and Biomass Assay

Cetyltrimethylammonium bromide (CTAB) method was used for genomic DNA extraction (Li et al., 2017). Briefly, 500 μl of CTAB buffer was added into crushed soybean hairy roots to lyse plant cells in a water bath at 60°C for 1 h, and an equal volume of chloroform-isoamyl alcohol (24:1) was added while vigorously shaking for 30 s. After centrifugation at 12,000 rpm at room temperature, the supernatant was transferred into a new Eppendorf tube, an equal volume of ice-cold 100% ethanol was added to precipitate DNA at –20°C for more than half an hour, and then 75% ethanol was used to wash DNA. After drying in the hood, 100 μl of sterilized H_2_O was added to dissolve DNA at 55°C. The extracted DNA was also quantified by Nanodrop and then diluted into the same concentration for biomass assay. Primers of *GmCYP2* (Hu et al., 2009) from soybean and *PsActin* from *P. sojae* were used for biomass assay (Shi et al., 2020; Supplementary Table 5).

### Immunoblotting Analyses

Proteins from the sample lysate were fractionated by SDS-PAGE gel. The separated proteins were transferred from gels to PVDF blotting membrane (GE, A10203127) (pretreated with methanol for 15 s) using Transfer Buffer (BIO-RAD, Cat. #10026938). The membrane was then blocked using 5% non-fat dry milk dissolved in TBST (TBS with 0.1% Tween 20) (also called TBSTM) for 1 h at room temperature with 30–40 rpm shaking, followed by three washes with TBST, and then primary antibody anti-Flag (1:5,000; MBL, M185-3L) and secondary antibody goat anti-mouse antibody (1:5,000; MBL, Lot 366) were applied to the membranes for 1 h in order. Finally, the membrane was visualized using the Western Blotting Substrate kit (Thermo Fisher Scientific, 34580) by multifunctional fluorescent molecular imager (GE, Amersham Imager 600) at 780 and 800 nm excitation (Chen et al., 2020b).

### Statistical Analysis

The statistical analysis in biomass assay, gene expression profile, and oospore numbers quantification was performed using an unpaired *T*-test in Graphpad Prism5.

## Data Availability Statement

The original contributions presented in the study are included in the article/Supplementary Material, further inquiries can be directed to the corresponding author/s.

## Author Contributions

YQ conceived and designed the experiments and revised the manuscript. SF, JS, YH, DL, and LG performed the experiments. ZZ and GL provided the suggestion for this research. SF and JS analyzed the experimental data and wrote the manuscript. All authors have read and approved the final manuscript.

## Conflict of Interest

The authors declare that the research was conducted in the absence of any commercial or financial relationships that could be construed as a potential conflict of interest.

## Publisher’s Note

All claims expressed in this article are solely those of the authors and do not necessarily represent those of their affiliated organizations, or those of the publisher, the editors and the reviewers. Any product that may be evaluated in this article, or claim that may be made by its manufacturer, is not guaranteed or endorsed by the publisher.

## Footnotes

1
https://www.soybase.org/

2
https://www.ncbi.nlm.nih.gov/

3
http://pfam.xfam.org/

4
https://itol.embl.de/

5
https://www.soybase.org/sgn/
