# Supplementary material for: Genome-Wide Analysis of Soybean Lateral Organ Boundaries Domain Gene Family Reveals the Role in Phytophthora Root and Stem Rot
Source: Front Plant Sci. 2022 May 4;13:865165. doi: 10.3389/fpls.2022.865165 (PMC9116278; doi:10.3389/fpls.2022.865165)
Supplement: Supplementary file 1 [file Data_Sheet_1.docx]

**Supplementary Table 1Ⅰ***LBD* genes identified in *C. sativus* in this study

| Gene identifier | Gene name | Genomic position |
| --- | --- | --- |
| KGN64334.1 | CsLBD1 | chr01:572928.573475 |
| XP_031737414.1 | CsLBD2 | chr01:692673.695698 |
| XP_004138241.2 | CsLBD3 | chr01:2368045.2369371 |
| XP_004147298.1 | CsLBD4 | chr01:8384563.8385641 |
| XP_004147299.1 | CsLBD5 | chr01:8399422.8400833 |
| XP_004145462.1 | CsLBD6 | chr01:32657236.32662446 |
| KAE8651792.1 | CsLBD7 | chr02:17410.18044 |
| KAE8652162.1 | CsLBD8 | chr02:1144321.1144627 |
| XP_004152887.1 | CsLBD9 | chr02:5808134.5810053 |
| XP_004148465.1 | CsLBD10 | chr02:14567535.14569405 |
| XP_011650178.1 | CsLBD11 | chr02:17560391.17560966 |
| XP_031737200.1 | CsLBD12 | chr02:22983031.22984125 |
| XP_004134346.1 | CsLBD13 | chr03:7849919.7851107 |
| XP_004140854.1 | CsLBD14 | chr03:12579286.12580125 |
| XP_004145641.1 | CsLBD15 | chr03:16836498.16838184 |
| XP_004152038.1 | CsLBD16 | chr03:19991891.19993387 |
| XP_004152037.2 | CsLBD17 | chr03:20013931.20015673 |
| XP_004136784.1 | CsLBD18 | chr03:33323742.33326690 |
| XP_004141196.1 | CsLBD19 | chr03:34700653.34703452 |
| XP_004141401.1 | CsLBD20 | chr04:24675755.24677024 |
| KAE8648523.1 | CsLBD21 | chr05:640631.645055 |
| KAE8648572.1 | CsLBD22 | chr05:990393.990530 |
| XP_004147712.1 | CsLBD23 | chr05:5004050.5005159 |
| XP_004148190.1 | CsLBD24 | chr05:6402432.6403197 |
| XP_004146476.1 | CsLBD25 | chr05:6752725.6755001 |
| XP_011655399.1 | CsLBD26 | chr05:22166498.22169470 |
| XP_004135401.1 | CsLBD27 | chr05:27325892.27325892 |
| XP_031741768.1 | CsLBD28 | chr05:29930284.29934135 |
| XP_004138415.1 | CsLBD29 | chr06:1330538.1331211 |
| XP_004138554.1 | CsLBD30 | chr06:1497009.1499050 |
| KAE8647756.1 | CsLBD31 | chr06:2898107.2898786 |
| XP_004143693.1 | CsLBD32 | chr06:4131720.4133238 |
| XP_004145058.1 | CsLBD33 | chr06:5177734.5180740 |
| XP_004145059.2 | CsLBD34 | chr06:5189719.5192981 |
| XP_004150042.1 | CsLBD35 | chr06:9817208.9819888 |
| XP_004143222.1 | CsLBD36 | chr06:11927494.11928535 |
| XP_004149555.1 | CsLBD37 | chr06:14608488.14610154 |
| XP_004141875.1 | CsLBD38 | chr06:25950356.25951626 |
| XP_004134907.3 | CsLBD39 | chr06:30345596.30346631 |
| XP_004139827.1 | CsLBD40 | chr07:11638763.11641523 |
| XP_004150342.1 | CsLBD41 | chr07:16618830.16619966 |
| XP_011659803.2 | CsLBD42 | chr07:21984733.21986615 |

**Supplementary Table 2Ⅰ***LBD* genes identified in *P. vulgaris* in this study

| Gene identifier | Gene name | Genomic position |
| --- | --- | --- |
| XP_007162000.1 | PvLBD1 | chr01:32377803.32381015 |
| XP_007162525.1 | PvLBD2 | chr01:41835988.41837718 |
| XP_007162526.1 | PvLBD3 | chr01:41858110.41859150 |
| XP_007163564.1 | PvLBD4 | chr01:50407242.50408649 |
| XP_007163576.1 | PvLBD5 | chr01：50490786.50491694 |
| XP_007156728.1 | PvLBD6 | chr02：1403702.1405256 |
| XP_007156831.1 | PvLBD7 | chr02：2305438.2308774 |
| XP_007153445.1 | PvLBD8 | chr03：3607760.3609168 |
| XP_007155464.1 | PvLBD9 | chr03：41772866.41775107 |
| XP_007155842.1 | PvLBD10 | chr03：45862977.45864555 |
| XP_007155957.1 | PvLBD11 | chr03:47230938.47232422 |
| XP_007155958.1 | PvLBD12 | chr03:47240211.47240741 |
| XP_007156006.1 | PvLBD13 | chr03:47782826.47783356 |
| XP_007150999.1 | PvLBD14 | chr04:999365.1001208 |
| XP_007151649.1 | PvLBD15 | chr04:8994761.8997674 |
| XP_007151760.1 | PvLBD16 | chr04:11301140.11304440 |
| XP_007152759.1 | PvLBD17 | chr04:43937823.43939653 |
| XP_007149195.1 | PvLBD18 | chr05:5784393.5788747 |
| XP_007150227.1 | PvLBD19 | chr05:36555652.36557505 |
| XP_007150515.1 | PvLBD20 | chr05:38437147.38437665 |
| XP_007146826.1 | PvLBD21 | chr06:19261111.19262127 |
| XP_007147329.1 | PvLBD22 | chr06:23022415.23023396 |
| XP_007143107.1 | PvLBD23 | chr07:3508488.3510206 |
| XP_007143674.1 | PvLBD24 | chr07:9386914.9388470 |
| XP_007143908.1 | PvLBD25 | chr07:14633239.14634591 |
| XP_007144924.1 | PvLBD26 | chr07:43334104.43335221 |
| XP_007144925.1 | PvLBD27 | chr07:43360166.43361521 |
| XP_007139150.1 | PvLBD28 | chr08:596737.598263 |
| XP_007139994.1 | PvLBD29 | chr08:7225971.7229473 |
| XP_007140816.1 | PvLBD30 | chr08:27548048.27550161 |
| XP_007140818.1 | PvLBD31 | chr08:27548048.27550161 |
| XP_007141403.1 | PvLBD32 | chr08:49887811.49888939 |
| XP_007141734.1 | PvLBD33 | chr08:53342368.53343134 |
| XP_007141747.1 | PvLBD34 | chr08:53458978.53459532 |
| XP_007142059.1 | PvLBD35 | chr08:56397459.56398768 |
| XP_007142160.1 | PvLBD36 | chr08:57021563.57022123 |
| XP_007142295.1 | PvLBD37 | chr08:57893659.57894482 |
| XP_007136211.1 | PvLBD38 | chr09:6159747.6161521 |
| XP_007137607.1 | PvLBD39 | chr09:20639950.20641396 |
| XP_007138029.1 | PvLBD40 | chr09:25537328.25539743 |
| XP_007134896.1 | PvLBD41 | chr10:31635596.31639916 |
| XP_007135547.1 | PvLBD42 | chr10:41055901.41057959 |
| XP_007131989.1 | PvLBD43 | chr11:4940411.4940811 |
| XP_007132174.1 | PvLBD44 | chr11:6484393.6486336 |
| XP_007132691.1 | PvLBD45 | chr11:18195659.18196246 |
| XP_007155357.1 | PvLBD46 | chr11:18195659.18196246 |
| XP_007133379.1 | PvLBD47 | chr11:44798536.44804073 |
| XP_007133438.1 | PvLBD48 | chr11:45373639.45374829 |
| XP_007133649.1 | PvLBD49 | chr11:47427155.47428248 |
| AOR16520.1 | PvLBD50 | chr11:51156470.51157037 |

**Supplementary Table 3Ⅰ**Information of 16 plant species for *LBD* family analyses

| **General classification** | **Species** | **Gene numbers in LBD family** | **Polyploid** | **Data Source** | **Gene name Abbreviation** |
| --- | --- | --- | --- | --- | --- |
| **Green alga** | *Cylindrocystis brebissonii* | 6 | haploid | Cited(Zhang et al. 2020) | YOXI/RPGL |
| **Moss** | *Physcomitrella patens* | 30 | haploid | Cited(Zhang et al. 2020) | Pp |
| **Fern** | *Selaginella moellendorffii* | 24 | Haploid or diploid | Cited(Zhang et al. 2020) | SELMO |
| **Basal angiosperm** | *Amborella trichopoda* | 20 | - | Cited(Zhang et al. 2020) | AMTR |
| **Dicotyledon** | *Arabidopsis thaliana* | 42 | diploid | Cited(Shuai et al. 2002) | AtLBD |
|  | *Glycine max* | 90 | tetraploid | Cited(Yang et al. 2017) | GmLBD |
|  | *Phaseolus vulgaris* | 50 | diploid | NCBI Database | PvLBD |
|  | *Solanum tuberosum* | 39 | tetraploid | Cited(Zhang et al. 2020) | PGSC |
|  | *Cucumis sativus* | 42 | diploid | NCBI Database | CsLBD |
|  | *Medicago truncatula* | 57 | diploid | Cited(Zhang et al. 2020) | MTR |
|  | *Gossypium raimondii* | 65 | tetraploid | Cited(Zhang et al. 2020) | B456 |
|  | *Brassica napus* | 118 | tetraploid | Cited(Zhang et al. 2020) | Bna |
| **Monocotyledon** | *Oryza sativa* | 37 | diploid | Cited(Zhang et al. 2020) | BGIOSGA |
|  | *Sorghum bicolor* | 33 | diploid | Cited(Zhang et al. 2020) | SORBI |
|  | *Zea mays* | 49 | diploid | Cited(Zhang et al. 2020) | Zm00001d |
|  | *Triticum aestivum* | 86 | hexaploid | Cited(Zhang et al. 2020) | TraesCS |

**Supplementary Table 4** Sources of 19 *GmLBD* candidate genes

| **Species** | **LBD genes related with plant immunity** | | **Homologues in Soybean** | **Accession number** |
| --- | --- | --- | --- | --- |
| ***A. thaliana*** | AtLBD20 | At3g03760 | GmLBD6 | Glyma.02G166400.1 |
|  |  |  | GmLBD81 | Glyma.19G122500.1 |
|  |  |  | GmLBD42 | Glyma.10G087200.1 |
| ***C. sinensis*** | CsLOB1 | orange1.1g026556m | GmLBD43 | Glyma.10G213500.1 |
|  | CsLOB2 | orange1.1g045080m | GmLBD49 | Glyma.12G072000.1 |
|  | CsLOB3 | orange1.1g047491m | GmLBD55 | Glyma.13G328500.1 |
| ***G. max*** | GmLBD9 | Glyma.02G262400.1 |  |  |
|  | GmLBD16 | Glyma.04G044800.1 |  |  |
|  | GmLBD23 | Glyma.05G103300.1 |  |  |
|  | GmLBD30 | Glyma.07G091600.1 |  |  |
|  | GmLBD31 | Glyma.07G223600.1 |  |  |
|  | GmLBD37 | Glyma.08G365100.1 |  |  |
|  | GmLBD45 | Glyma.11G049300.1 |  |  |
|  | GmLBD51 | Glyma.13G046300.1 |  |  |
|  | GmLBD59 | Glyma.14G057600.1 |  |  |
|  | GmLBD70 | Glyma.17G162000.1 |  |  |
|  | GmLBD88 | Glyma.20G177600.1 |  |  |
|  | GmLBD90 | Glyma.U020900.1 |  |  |
|  | GmLBD63 | Glyma.15G045200.1 |  |  |

**Supplementary Table 5Ⅰ**Primer pairs used in this study

| **Gene Names** | **Sense primers (5'-3')** | **Antisense primers (5'-3')** | **Function** |
| --- | --- | --- | --- |
| GmLBD9 | TCAACTTCCTCTGTTTTAAGTT | AACGGTTATGTTTGGGAC | QRT-PCR |
| GmLBD16 | GTTATTTAATTATGAGCGGAGAG | TGGAACTAGAATATCGAAAGG | QRT-PCR |
| GmLBD23 | TTCGCAGAAGGTTGAG | TCGAAGAAACATATAGGAGAGT | QRT-PCR |
| GmLBD30 | ATGTTTTTTTTAATCTTCACCC | CAAACAATTTTCACAATGTATATG | QRT-PCR |
| GmLBD31 | AATCCTCCTCCTCCTTC | TAATTATAGTGTTAATTTCCCTTCT | QRT-PCR |
| GmLBD37 | GTCATCAATTTCGCGTC | CGACCATAAAACTTAGCGA | QRT-PCR |
| GmLBD43 | TTTGCTCTGTTTTCCTCTGTTTCT | GAAGGTGTTGATGTTGTGGTTTTC | QRT-PCR |
| GmLBD45 | GATCTCCGTCGATGAAC | CTCAAACACGAAACTTAGAGA | QRT-PCR |
| GmLBD49 | GTTCCTAAAAAACACACAGCA | GGAGGTGAAGGTGGTGTT | QRT-PCR |
| GmLBD51 | TGAGAAGTTTAGAAGCACTTG | GTTCCATTTGCAACAAATAC | QRT-PCR |
| GmLBD55 | TAGTCTCTTTGCACAAGGT | ATCAGTGCTTAGAAACATACTG | QRT-PCR |
| GmLBD59 | TACCACCACTTCTGATGC | GGGGTCATAACTAACAACATATC | QRT-PCR |
| GmLBD63 | CCACAGGAATATTCACCA | ATTGCCTTGTATAATTCTGG | QRT-PCR |
| GmLBD70 | GTTGATTCAAACATAAACACAC | GCTTATTTTTTGTTCTTGTTATTA | QRT-PCR |
| GmLBD88 | ATATTTGCATCAGAGAGATT | TACCATATAACTAATTAAATGACTTT | QRT-PCR |
| GmLBD90 | CAAAGGCACAAACCCTC | GTGTGTTTTAGGGACACAAAG | QRT-PCR |
| 5941-GmLBD9 | TTACAATTACCATGGGGCGCGCCATGGGTGGAAATTCCCCT | CTCTCTAGACTCACCTAGGATCCTCAGGTACCGGGCCCCC | overexpression in hairy soybean |
| 5941-GmLBD16 | TTACAATTACCATGGGGCGCGCCATGGTCTTCCTCTATGAT | CTCTCTAGACTCACCTAGGATCCTCAGGTACCGGGCCCCC | overexpression in hairy soybean |
| 5941-GmLBD23 | TTACAATTACCATGGGGCGCGCCATGAGTTGCAACGGGTGC | CTCTCTAGACTCACCTAGGATCCTCAGGTACCGGGCCCCC | overexpression in hairy soybean |
| 5941-GmLBD88 | TTACAATTACCATGGGGCGCGCCATGGAGTACAAAGTGAAA | CTCTCTAGACTCACCTAGGATCCTCAGGTACCGGGCCCCC | overexpression in hairy soybean |
| QBV3-GmLBD9 | AAAAAAGCAGGCTCAGGGGATATCACTCTTTGATTTGTATTC | GAAAGCTGGGTGCAGGGCGATATCTTTTACTTTTTTGGAAAC | RNAi in hairy soybean |
| QBV3-GmLBD16 | AAAGCAGGCTCAGGGGATATCACAAGCTAAGTTGGAATA | AGCTGGGTGCAGGGCGATATCGATCGAAGAAAAAGACGG | RNAi in hairy soybean |
| QBV3-GmLBD23 | AAAAAAGCAGGCTCAGGGGATATCGCAGAATTAAACACACAC | GAAAGCTGGGTGCAGGGCGATATCGCTTATTTTTTGTTGTTA | RNAi in hairy soybean |
| QBV3-GmLBD88 | AAAGCAGGCTCAGGGGATATCACAACAGTTTTGGTACTG | AGCTGGGTGCAGGGCGATATCTAACTAATTAAATGACTT | RNAi in hairy soybean |
