# Supplementary material for: Genome-Wide Analysis of Soybean Lateral Organ Boundaries Domain Gene Family Reveals the Role in Phytophthora Root and Stem Rot
Source: Front Plant Sci. 2022 May 4;13:865165. doi: 10.3389/fpls.2022.865165 (PMC9116278; doi:10.3389/fpls.2022.865165)
Supplement: Supplementary file 2 [file Presentation_1.pptx]

## Slide 1
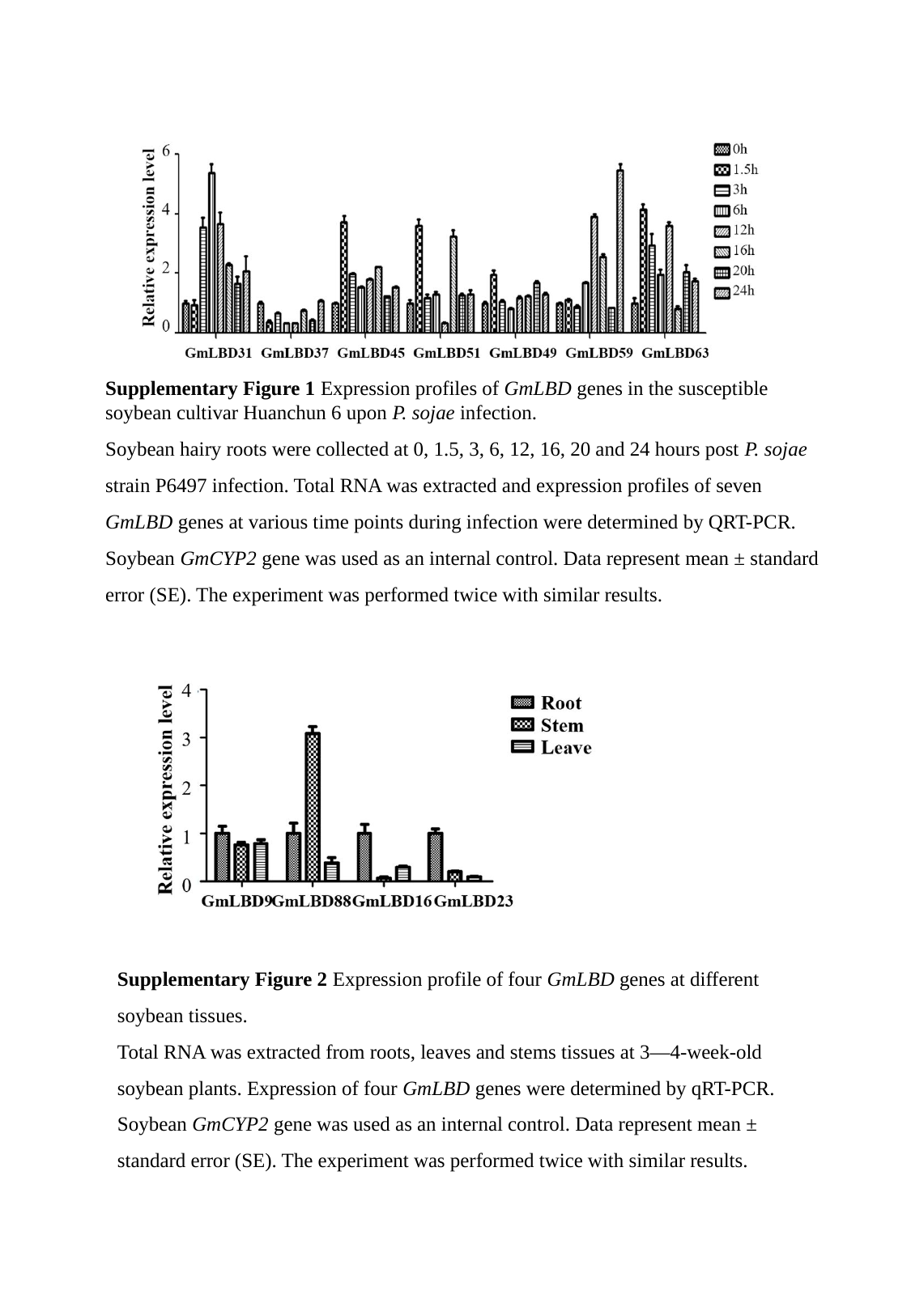

Supplementary Figure 1 Expression profiles of GmLBD genes in the susceptible soybean cultivar Huanchun 6 upon P. sojae infection.
Soybean hairy roots were collected at 0, 1.5, 3, 6, 12, 16, 20 and 24 hours post P. sojae strain P6497 infection. Total RNA was extracted and expression profiles of seven GmLBD genes at various time points during infection were determined by QRT-PCR. Soybean GmCYP2 gene was used as an internal control. Data represent mean ± standard error (SE). The experiment was performed twice with similar results.
Supplementary Figure 2 Expression profile of four GmLBD genes at different soybean tissues.
Total RNA was extracted from roots, leaves and stems tissues at 3—4-week-old soybean plants. Expression of four GmLBD genes were determined by qRT-PCR. Soybean GmCYP2 gene was used as an internal control. Data represent mean ± standard error (SE). The experiment was performed twice with similar results.
